# Supplementary material for: Large-scale statistical dissection of sequence-derived biochemical features distinguishing soluble and insoluble proteins
Source: PLoS One. 2026 Jun 5;21(6):e0344883. doi: 10.1371/journal.pone.0344883 (PMC13240910; doi:10.1371/journal.pone.0344883)
Supplement: Attachment — Submitted filename: Response to Reviewers.pdf [file pone.0344883.s006.pdf]

LARGE-SCALE STATISTICAL DISSECTION OF SEQUENCE-DERIVED BIOCHEMICAL FEATURES DISTINGUISHING SOLUBLE AND INSOLUBLE PROTEINS

Dear Reviewers and Editors of the PLOS One,

We would like to thank you so much, the reviewers and editors, for your careful and thorough reading of this manuscript and for your thoughtful comments and constructive suggestions, which significantly improved its quality. Our responses are in *italics* following the reviewers' comments, and new changes are *highlighted in yellow* in our manuscript.
Yours sincerely,

Authors

Dear Reviewer 1,

We would like to answer your comments as follows:

1. **Incorrect reference numbering: The citation format is inconsistent throughout the manuscript. For example, on page 1, line 4, the reference is cited as [1, 6], whereas it should appear sequentially (e.g., [1, 2]). The authors are advised to use reference management software such as EndNote, Zotero, or similar tools to correct and standardize citations across the entire manuscript.**

Our answer: *Thank you very much for this valuable comment, and please be aware that we have revised the manuscript accordingly and corrected the reference numbering as suggested by the reviewer.*

2. **Lack of sequence data transparency: The authors state that a total of 36 sequence-derived biochemical features were computed for each protein. However, the manuscript does not provide the number of FASTA files used or the actual protein sequences. This information should be included or properly referenced.**

Our answer: *Thank you very much for this valuable comment, and please kindly note that the revised manuscript has been updated based on the reviewers' comments.*

The revised Dataset section now explicitly states that our analysis used the three original FASTA files from the benchmark dataset reported by Zhang et al. (2024). We also clarify that these FASTA files were used without modification.

Furthermore, in the Data and Code Availability section, we now clearly indicate that the raw sequences and labels are publicly accessible through Zenodo. Additionally, the full feature matrix used for all statistical analyses, the processed data and analysis outputs is provided in the GitHub repository (see the Dataset subsection on page 3, and the Data and Code Availability section on pages 15–16)

3. **Missing supporting information: The manuscript refers to supporting information, but no supplementary materials are provided. All supporting data should be included and clearly linked.**

Our answer*: Thank you very much for this valuable comment, and please kindly note that the revised manuscript has been updated based on the reviewers' comments.*

All supporting information has now been included and clearly linked through a separate Supporting Information document submitted together with the revised manuscript. The supplementary materials include Tables S1–S5 and Figures S1–S4 (a PDF file attached).

4. **Weak conclusion section: The conclusion is not clearly articulated and should be rewritten. It should explicitly explain how the statistical analysis contributes to distinguishing between soluble and insoluble proteins and highlight the significance of the findings.**

Our answer: *Thank you very much for this important comment, and please kindly note that the revised manuscript's conclusion has been updated based on the reviewers' comments.*

We clarify how the statistical analysis contributes to distinguishing between soluble and insoluble proteins and make significance of these findings more explicit as well (see the Conclusion section on page 14).

5. **Introduction requires improvement: The introduction lacks clarity and coherence and should be thoroughly revised to better contextualize the study and its objectives.**

Our answer: *Thank you very much for this important comment. In the revised manuscript, we clarify the introduction more clarity and coherence as follows:*

The revised Introduction now provides a clearer explanation on pages 1–3.

Overall, please kindly note that the revised manuscript states that the purpose of this study is not to develop another high-capacity predictive model, but rather to perform a statistically rigorous large-scale analysis of 36 sequence-derived biochemical features in order to quantify their effect sizes, redundancy structure, and practical discriminatory contribution in distinguishing soluble and insoluble proteins. Additionally, this study also presents a simple baseline model intended to serve as a lower-bound reference for evaluating more complex predictive approaches in future work

Dear Reviewer 2,

We would like to answer your comments as follows:

1. **To ensure no bias in the AUC and MCC comparisons, it is suggested that a clarification be provided if the reference models were evaluated on the exact same merged dataset or if the performance that is reported is taken straight from the originally published data**

Our answer: *Thank you very much for this valuable comment, and please be aware that we have revised the manuscript as suggested by the reviewer. Specifically, in the Results section (page 12), we now clearly confirm that the performance metrics (AUC and MCC) were taken directly from Zhang et al. (2024) (https://doi.org/10.1093/bib/bbae404).*

2. **In the discussion section, negative charge appearing more influential than positive charge can be elaborated upon more. Is this a general biophysical principle of the proteome or if it is a bias in the E. coli based recombinant expression data used?**

Our answer: *Thank you very much for this valuable comment, and please kindly note that we have revised the Discussion section accordingly based on the reviewers' comments as follows on page 9. The observed asymmetry likely reflects both general biophysical effects and E. coli expression-system bias and the dataset is based on recombinant expression in E. coli, therefore, generalization should be made with caution.*

3. **It is suggested that in the conclusions section including a comment on whether the weak signal interactions are additive or if they involve higher-order sequence patterns that classical descriptors cannot capture.**

Our answer*: Thank you very much for this valuable comment, and please kindly note that the Conclusion section has been updated accordingly to clarify that based on the reviewers' comments as follows on page 14-15.*

4. **The ROC curves in figure 2 shows a significant overlap. Adding a density plot for say like the top two orthogonal features to demonstrate the extensive overlap as mentioned in the paper would be helpful in demonstrating to the reader the difficulty of the classification process.**

Our answer: *Thank you very much for this important comment, and please kindly note that the Results section has been updated based on the reviewers' comments as follows on page 10-11.*

We added Figure 4 (the density distributions of the two features). We also clarified that this overlap explains the limited univariate ROC performance and highlights the difficulty of the classification process.

Dear Reviewer 3,

We would like to answer your comments as follows:

Reviewer's statement: The manuscript presents a statistical analysis of sequence-derived features associated with protein solubility. While the study is carefully conducted and the dataset appears comprehensive, the overall contribution remains limited in terms of novelty and impact. Most of the analyzed features (e.g., amino acid composition, sequence length, and charge properties) are well-established determinants of solubility and have been extensively explored in previous studies.

Our answer: *Thank you very much for this valuable comment. Please note that the manuscript has been thoroughly revised to more explicitly highlight its innovative contribution, its necessity, and its clear distinction from previous literature/studies.*

The novelty of the present work does not lie in introducing new descriptors. Rather, it lies in providing a large-scale, statistically controlled quantification of their practical effect sizes, redundancy structure, and descriptive discriminative limits in a large publicly available dataset, from there establishing an interpretable baseline for what can or cannot be captured by classical global sequence-derived features. This point is now stated more explicitly in the Introduction (pp. 2–3).

We have also reiterated this contribution more clearly in the Conclusion (pp. 14–15), where we clarify that the study refines the interpretation of widely accepted solubility determinants by showing that their practical effect sizes are substantially smaller than statistical significance alone may suggest. Also, the proposed framework should be understood as a transparent, lower-bound, and interpretable reference for evaluating the added value of more complex models, rather than as a competitive predictive model.

We confirm that every change has been made in the revised manuscript (v1), a high level of refinement in language aspect, with yellow highlighting for easy verification. We are confident that the manuscript has been substantially improved thanks to your valuable feedback.

Thank you once again for your time and constructive comments. We look forward to your positive decisions.

Sincerely yours,
