# Supplementary material for: Large-scale statistical dissection of sequence-derived biochemical features distinguishing soluble and insoluble proteins
Source: PLoS One. 2026 Jun 5;21(6):e0344883. doi: 10.1371/journal.pone.0344883 (PMC13240910; doi:10.1371/journal.pone.0344883)
Supplement: Attachment — Submitted filename: Re-review comments.pdf [file pone.0344883.s005.pdf]

All queries have been addressed carefully by the author. However, the conclusion section is overly long and lacks clarity, making it difficult to understand the key takeaways of the study. I recommend that the manuscript be accepted only after the conclusion is revised to be more concise and clearly summarize the main findings.
