# Supplementary material for: Large-scale statistical dissection of sequence-derived biochemical features distinguishing soluble and insoluble proteins
Source: PLoS One. 2026 Jun 5;21(6):e0344883. doi: 10.1371/journal.pone.0344883 (PMC13240910; doi:10.1371/journal.pone.0344883)
Supplement: Attachment — Submitted filename: Re-review 2 comments.pdf [file pone.0344883.s007.pdf]

The authors have adequately addressed all the queries. I recommend that the editor accept the manuscript without any further revisions.
