# Supplementary material for: Large-scale statistical dissection of sequence-derived biochemical features distinguishing soluble and insoluble proteins
Source: PLoS One. 2026 Jun 5;21(6):e0344883. doi: 10.1371/journal.pone.0344883 (PMC13240910; doi:10.1371/journal.pone.0344883)
Supplement: Attachment — Submitted filename: Response to Reviewers.docx [file pone.0344883.s008.docx]

# LARGE-SCALE STATISTICAL DISSECTION OF SEQUENCE-DERIVED BIOCHEMICAL FEATURES DISTINGUISHING SOLUBLE AND INSOLUBLE PROTEINS

# *Dear Reviewers and Editors of the PLOS One,*

We would like to thank you so much, the reviewers and editors, for your careful and thorough reading of this manuscript and for your thoughtful comments and constructive suggestions, which significantly improved its quality. Our responses are in *italics* following the reviewers’ comments, and new changes are *highlighted in yellow* in our manuscript.

Yours sincerely,

Authors

***Dear Reviewer 1,***

We would like to answer your comments as follows: “**All queries have been addressed carefully by the author. However, the conclusion section is overly long and lacks clarity, making it difficult to understand the key takeaways of the study. I recommend that the manuscript be accepted only after the conclusion is revised to be more concise and clearly summarize the main findings.**”

***Our answer:*** *Thank you very much for this valuable comment, and please be aware that we have revised the manuscript’s conclusion accordingly as suggested by the reviewer. We greatly appreciate the reviewer’s recommendation.*

***Dear Reviewer 2,***

*Thank you very much for this valuable comment, we greatly appreciate the reviewer’s recommendation.*

***Dear Reviewer 3,***

*Thank you very much for this valuable comment, we greatly appreciate the reviewer’s recommendation.*

We confirm that every change has been made in the revised manuscript (v2), a high level of refinement in language aspect, with yellow highlighting for easy verification. We are confident that the manuscript has been substantially improved thanks to your valuable feedback.

Thank you once again for your time and constructive comments. We look forward to your positive decisions.

Sincerely yours,
