# Supplementary material for: Large-scale statistical dissection of sequence-derived biochemical features distinguishing soluble and insoluble proteins
Source: PLoS One. 2026 Jun 5;21(6):e0344883. doi: 10.1371/journal.pone.0344883 (PMC13240910; doi:10.1371/journal.pone.0344883)
Supplement: Attachment — Submitted filename: Reviewer comment.pdf [file pone.0344883.s003.pdf]

I recommend that the editor request major revisions for this manuscript. The authors should address the following concerns:

1. Incorrect reference numbering**:**
   The citation format is inconsistent throughout the manuscript. For example, on page 1, line 4, the reference is cited as [1, 6], whereas it should appear sequentially (e.g., [1, 2]). The authors are advised to use reference management software such as EndNote, Zotero, or similar tools to correct and standardize citations across the entire manuscript.
2. Lack of sequence data transparency:
   The authors state that a total of 36 sequence-derived biochemical features were computed for each protein. However, the manuscript does not provide the number of FASTA files used or the actual protein sequences. This information should be included or properly referenced.
3. Missing supporting information:
   The manuscript refers to supporting information, but no supplementary materials are provided. All supporting data should be included and clearly linked.
4. Weak conclusion section:
   The conclusion is not clearly articulated and should be rewritten. It should explicitly explain how the statistical analysis contributes to distinguishing between soluble and insoluble proteins and highlight the significance of the findings.
5. Introduction requires improvement:
   The introduction lacks clarity and coherence and should be thoroughly revised to better contextualize the study and its objectives.
